# Class effects of SGLT2 inhibitors on cardiorenal outcomes

**DOI:** 10.1186/s12933-019-0903-4

**Published:** 2019-08-05

**Authors:** Aaron Y. Kluger, Kristen M. Tecson, Andy Y. Lee, Edgar V. Lerma, Janani Rangaswami, Norman E. Lepor, Michael E. Cobble, Peter A. McCullough

**Affiliations:** 1 Baylor Heart and Vascular Institute, 621 N. Hall #H030, Dallas, TX 75226 USA; 2 Baylor Scott & White Research Institute, Dallas, TX USA; 3 Texas A&M College of Medicine Health Science Center, Dallas, TX USA; 4 Baylor University Medical Center, Dallas, TX USA; 5 Baylor Heart and Vascular Hospital, Dallas, TX USA; 6 UIC/Advocate Christ Medical Center, Oak Lawn, IL USA; 7 Einstein Medical Center, Philadelphia, PA USA; 8 Sidney Kimmel College of Thomas Jefferson University, Philadelphia, PA USA; 9 David Geffen School of Medicine at UCLA, Los Angeles, CA USA; 10 Cedars-Sinai Medical Center, Los Angeles, CA USA; 11 University of Utah School of Medicine, Salt Lake City, UT USA

**Keywords:** SGLT2 inhibitor, Empagliflozin, Canagliflozin, Dapagliflozin, CANVAS, EMPA–REG OUTCOME, DECLARE-TIMI 58, CREDENCE, Cardiovascular outcome trials, Heart failure hospitalization, Cardiovascular death, Albuminuria, Estimated glomerular filtration function, Chronic kidney disease, End-stage renal disease, Mortality

## Abstract

**Background:**

To summarize the four recent sodium-glucose cotransporter 2 inhibitor (SGLT2i) trials: Dapagliflozin Effect on CardiovascuLAR Events (DECLARE-TIMI 58), CANagliflozin CardioVascular Assessment Study (CANVAS) Program, Empagliflozin Cardiovascular Outcome Event Trial in Type 2 Diabetes Mellitus Patients–Removing Excess Glucose (EMPA–REG OUTCOME), Canagliflozin and Renal Events in Diabetes with Established Nephropathy Clinical Evaluation (CREDENCE), and explore the potential determinants for their cardiovascular, renal, and safety outcomes.

**Results:**

The composite renal outcome event rates per 1000 patient-years for drug and placebo, as well as the corresponding relative risk reductions, were 3.7, 7.0, 47%; 5.5, 9.0, 40%; 6.3, 11.5, 46%; 43.2, 61.2, 30% for DECLARE-TIMI 58, CANVAS, EMPA–REG OUTCOME, and CREDENCE, respectively (event definitions varied across trials). The major adverse cardiovascular (CV) event rates per 1000 patient-years for drug and placebo, as well as the corresponding relative risk reductions, were 22.6, 24.2, 7%; 26.9, 31.5, 14%; 37.4, 43.9, 14%; 38.7, 48.7, 20% for DECLARE-TIMI 58, CANVAS, EMPA–REG OUTCOME, and CREDENCE, respectively. DECLARE-TIMI 58 had the fewest cardiorenal events and CREDENCE the most. These differences were presumably due to varying inclusion criteria resulting in DECLARE-TIMI 58 having the best baseline renal filtration function and CREDENCE the worst (mean estimated glomerular filtration rate 85.2, 76.5, 74, 56.2 mL/min/1.73 m^2^ for DECLARE-TIMI 58, CANVAS, EMPA–REG OUTCOME, and CREDENCE, respectively). Additionally, CREDENCE had considerably higher rates of albuminuria (median urinary albumin-creatinine ratios (UACR) were 927, 12.3, and 13.1 mg/g for CREDENCE, CANVAS, and DECLARE-TIMI 58, respectively; EMPA–REG OUTCOME had 59.4% UACR < 30, 28.6% UACR > 30–300, 11.0% UACR > 300 mg/g).

**Conclusions:**

Dapagliflozin, empagliflozin, and canagliflozin have internally and externally consistent and biologically plausible class effects on cardiorenal outcomes. Baseline renal filtration function and degree of albuminuria are the most significant indicators of risk for both CV and renal events. Thus, these two factors also anticipate the greatest clinical benefit for SGLT2i.

## Background

Type 2 diabetes mellitus (T2DM) is significantly associated with cardiovascular disease (CVD) and is a risk factor for heart failure (HF); diabetic patients are hospitalized for HF approximately four times more frequently than nondiabetic patients [1–5]. T2DM is a risk factor for chronic kidney disease (CKD) and end-stage renal disease (ESRD) [6, 7]. T2DM is also associated with non-healing lower extremity wounds, deep tissue osteomyelitis, metabolic bone disease, anemia, pancreatitis, and diabetic ketoacidosis [8–10]. Further, T2DM medications often have deleterious side effects. Thiazolidinediones are linked to edema, HF hospitalization (HHF) and cardiovascular (CV) death in certain patient subsets [11–13]. Oral sulfonylureas are associated with hypoglycemia, myocardial infarction (MI), stroke, and CV death, although a recent intervention trial found that sulfonylureas had similar rates of CV events compared to pioglitazone (1.5/100 patient-years for both groups, hazard ratio (HR) = 0.96, 95% confidence interval (CI) 0.74–1.26, p = 0.79) [14–16].

The 2008 United States Food and Drug Administration (FDA) antidiabetic drug guidance required cardiovascular outcome trials (CVOTs) for novel antihyperglycemic medications to demonstrate that new drugs would not increase the risk for MI, stroke, or CV death [17]. The FDA has approved four sodium-glucose cotransporter 2 inhibitors (SGLT2i) based on these guidelines: canagliflozin (Invokana), dapagliflozin (Farxiga), empagliflozin (Jardiance), and ertugliflozin (Steglatro). A fifth SGLT2i, sotagliflozin (Zynquista), is in late clinical development. Multiple expert consensus decisions attest to the potential of SGLT2i as a promising new class for the treatment of patients with T2DM and established CVD [18, 19].

Three SGLT2i (canagliflozin, empagliflozin, dapagliflozin) have been studied in CVOTs; canagliflozin has also been studied in an additional randomized clinical trial involving patients with diabetic kidney disease [20–23]. This review will explore the design and results of each of the four key SGLT2i trials and discuss the potential determinants for their CV, renal, and safety outcomes.

## Methods

We reviewed the relevant trials’ original methodology and results papers. The methodological details and outcomes of the trials will be reviewed in the Results section below. As some p-values were not provided in all trials, we calculated them from the HR and 95% CI [24]. We calculated the relative risk reduction percentages from the HR. Continuous variables are presented as mean ± standard deviation or median [quartile 1, quartile 3], if skewed. Categorical variables are presented as frequency (%).

## Results

### The *EMPA–REG OUTCOME* Trial

The first SGLT2i CVOT, the Empagliflozin Cardiovascular Outcome Event Trial in Type 2 Diabetes Mellitus Patients–Removing Excess Glucose randomized double-blind controlled trial (EMPA–REG OUTCOME) assigned 7020 patients with T2DM and CVD to 10 mg or 25 mg of empagliflozin or placebo daily over a 3.1 year mean and median follow-up period [20, 25]. Study patients were required to have estimated glomerular filtration rate (eGFR) > 30 mL/min/1.73 m^2^ [calculated using the Modification of Diet in Renal Disease (MDRD) equation]; empagliflozin is indicated for T2DM patients with eGFR ≥ 45 mL/min/1.73 m^2^ [26].

**Fig. 1:**
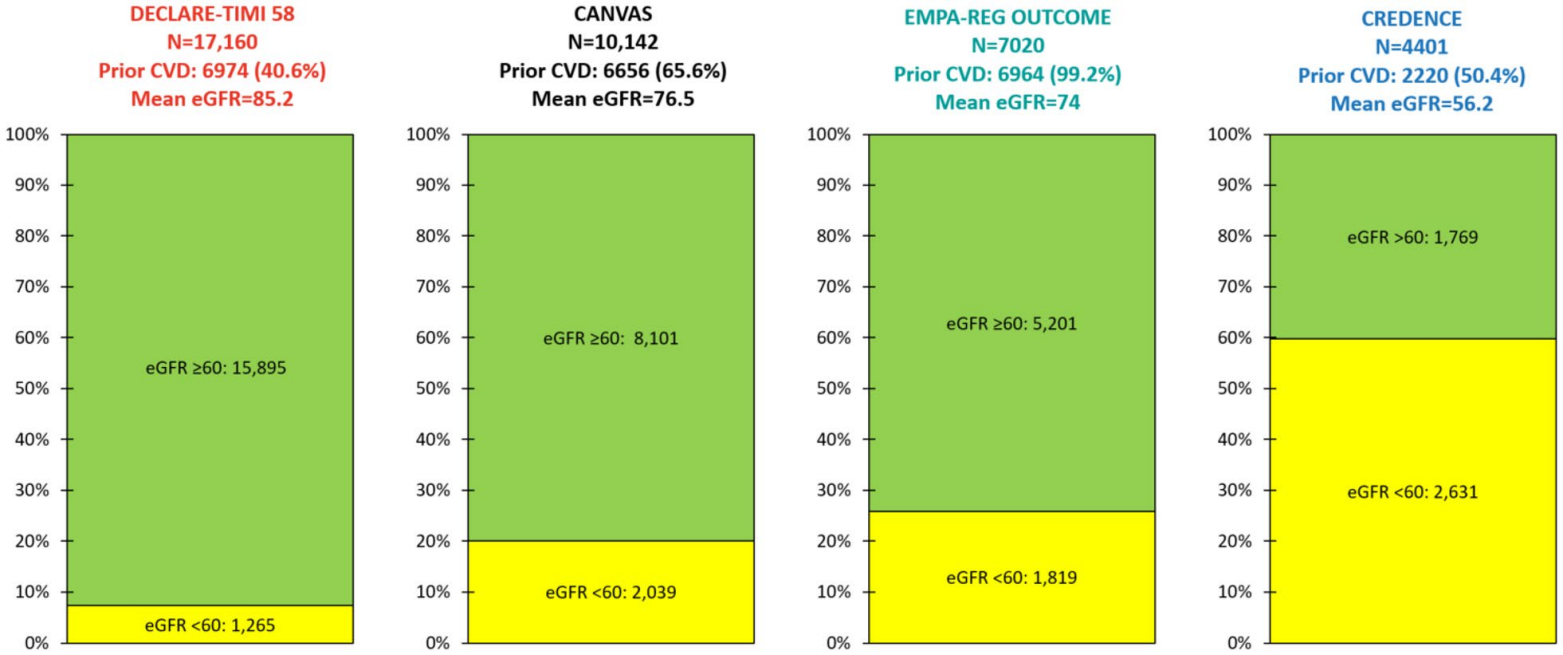
Baseline estimated glomerular filtration rates (eGFRs) and prior cardiovascular disease (CVD) rates in the Dapagliflozin Effect on CardiovascuLAR Events (DECLARE-TIMI 58), CANagliflozin CardioVascular Assessment Study (CANVAS) Program, Empagliflozin Cardiovascular Outcome Event Trial in Type 2 Diabetes Mellitus Patients–Removing Excess Glucose (EMPA–REG OUTCOME), and Canagliflozin and Renal Events in Diabetes with Established Nephropathy Clinical Evaluation (CREDENCE) trials. Prior CVD displayed as incidence (percentage)

Nearly all [6964 (99.2%)] EMPA–REG OUTCOME patients had established CVD, most commonly stable coronary artery disease (Fig. 1). The mean eGFR was 74 ± 21 mL/min/1.73 m^2^, 1819 (25.9%) patients had eGFR < 60 mL/min/1.73 m^2^, and 5201 (74.1%) had eGFR > 60 mL/min/1.73 m^2^ [20, 27]. There were 4171 (59.4%) patients with urinary albumin-creatinine ratio (UACR) < 30 mg/g, 2013 (28.7%) with UACR > 30–300 mg/g, and 769 (11.0%) with UACR > 300 mg/g. Although angiotensin-converting-enzyme inhibitor (ACEi) or angiotensin-receptor blocker (ARB) use was not required, they were used in 5666 (80.7%) patients.

**Fig. 2:**
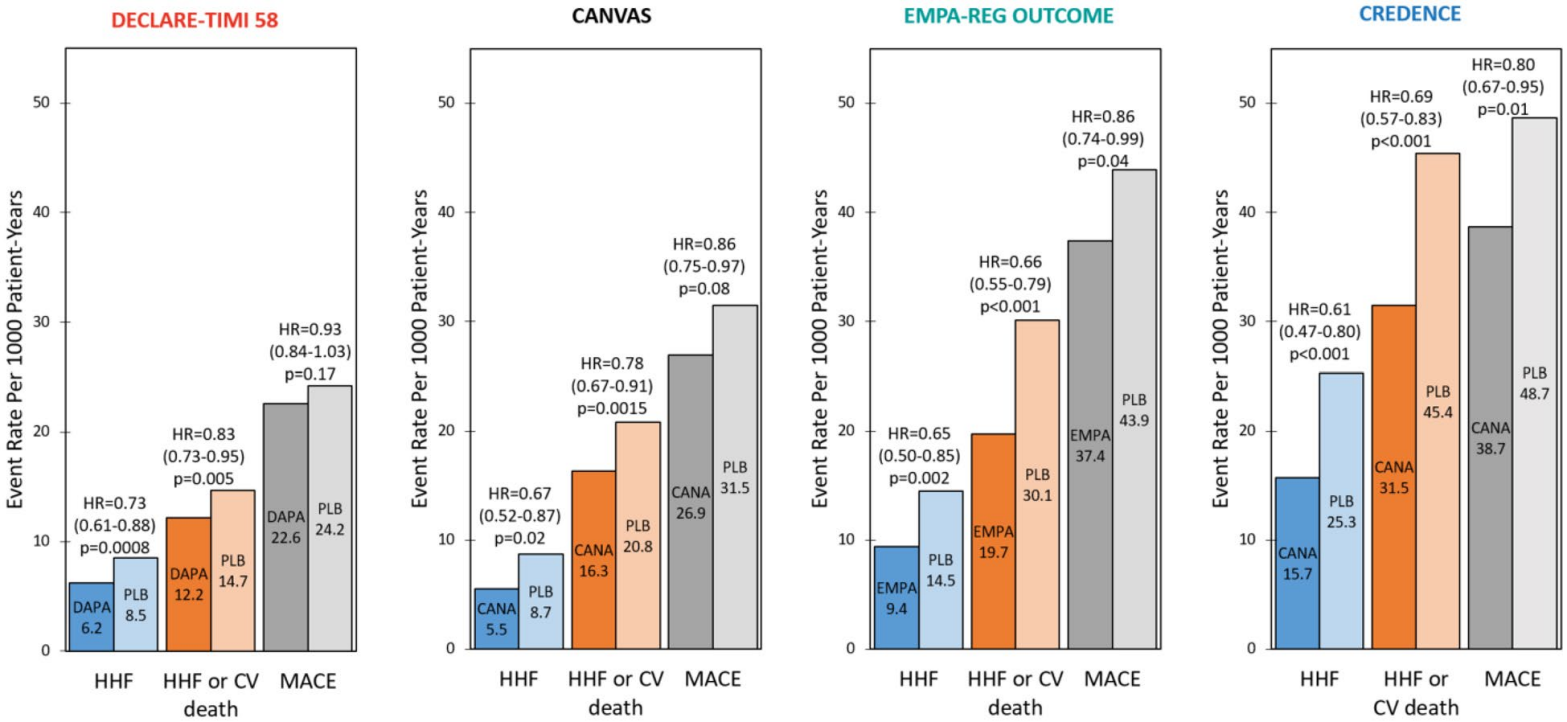
Heart failure hospitalization (HHF), HHF and cardiovascular (CV) death, and major adverse cardiovascular event (MACE) event rates per 1000 patients in the Dapagliflozin Effect on CardiovascuLAR Events (DECLARE-TIMI 58), CANagliflozin CardioVascular Assessment Study (CANVAS) Program, Empagliflozin Cardiovascular Outcome Event Trial in Type 2 Diabetes Mellitus Patients–Removing Excess Glucose (EMPA–REG OUTCOME), and Canagliflozin and Renal Events in Diabetes with Established Nephropathy Clinical Evaluation (CREDENCE) trials. Statistical outcomes displayed as hazard ratio, 95% confidence interval, p-value. *HR* hazard ratio, *DAPA* dapagliflozin, *CANA* canagliflozin, *EMPA* empagliflozin, *PLB* placebo

**Fig. 3:**
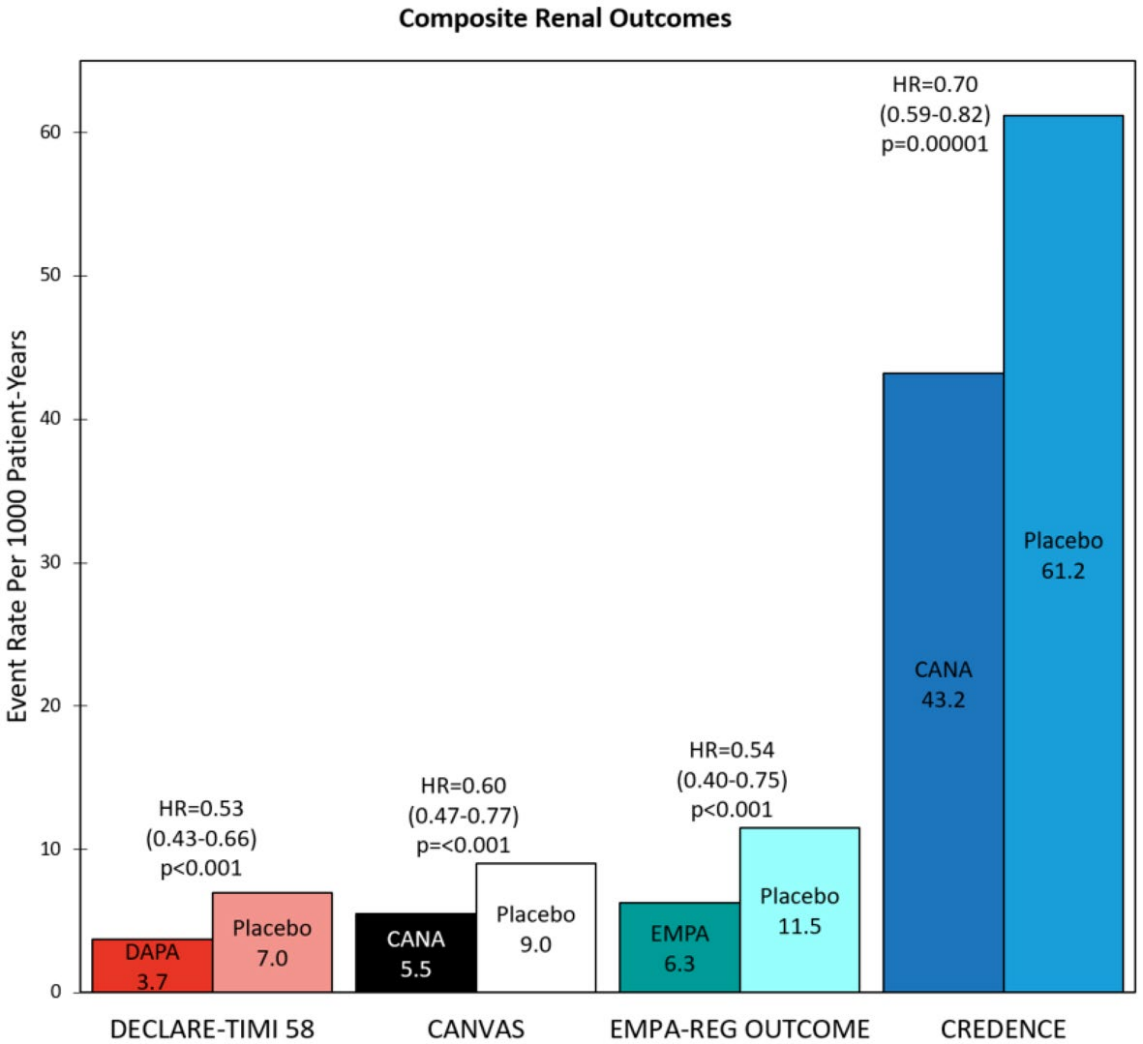
Composite renal outcome rates in the Dapagliflozin Effect on CardiovascuLAR Events (DECLARE-TIMI 58), CANagliflozin CardioVascular Assessment Study (CANVAS) Program, Empagliflozin Cardiovascular Outcome Event Trial in Type 2 Diabetes Mellitus Patients–Removing Excess Glucose (EMPA–REG OUTCOME), and Canagliflozin and Renal Events in Diabetes with Established Nephropathy Clinical Evaluation (CREDENCE) trials. Statistical outcomes displayed as hazard ratio, 95% confidence interval, p-value. Composite renal outcomes defined as follows: DECLARE-TIMI 58: ≥ 40% reduction in estimated glomerular filtration rate (eGFR) to < 60, end-stage renal disease (ESRD) (dialysis ≥ 90 days, transplant or sustained eGFR < 15), or renal/cardiovascular (CV) death; CANVAS: ≥ 40% reduction in eGFR, renal-replacement therapy (RRT) (transplant, chronic dialysis, or sustained eGFR < 15), or renal death; EMPA–REG OUTCOME: doubling of serum creatinine (Cr) with eGFR ≤ 45, RRT, or renal death; CREDENCE: doubling of serum Cr, ESRD (eGFR < 15, dialysis, or renal transplant), renal/CV death. *HR* hazard ratio, *DAPA* dapagliflozin, *CANA* canagliflozin, *EMPA* empagliflozin

The primary composite CV endpoint (CV death, nonfatal MI, or nonfatal stroke) occurred in 10.5% of empagliflozin patients compared to 12.1% of placebo patients (rate per 1000 patient-years = 37.4 vs. 43.9, respectively; HR = 0.86, 95% CI 0.74–0.99, p = 0.04) (Fig. 2). With regard to secondary outcomes, HHF occurred in 2.7% of empagliflozin patients compared to 4.1% of placebo patients (rate per 1000 patient-years = 9.4 vs. 14.5, HR = 0.65, 95% CI 0.50–0.85, p = 0.002). HHF or CV death (excluding fatal stroke) occurred in 5.7% of empagliflozin patients compared to 8.5% of placebo patients (rate per 1000 patient-years = 19.7 vs. 30.1, HR = 0.66, 95% CI 0.55–0.79, p < 0.001). The composite renal outcome [doubling of serum creatinine level accompanied by an eGFR ≤ 45 mL/min/1.73 m^2^, initiation of renal-replacement therapy (RRT), or renal death] occurred in 1.7% of empagliflozin patients compared to 3.1% of placebo patients (rate per 1000 patient-years = 6.3 vs. 11.5, HR = 0.54, 95% CI 0.40–0.75, p < 0.001) (Fig. 3) [28].

### The *CANVAS* program

The second SGLT2i CVOT, the CANagliflozin CardioVascular Assessment Study (CANVAS) Program combined the CANVAS and CANVAS-Renal (CANVAS-R) study cohorts into a randomized double-blind controlled trial, assigning 10,142 T2DM patients to daily canagliflozin (100 mg with optional increase to 300 mg) or placebo over a 2.4 year median (188.2 week mean) follow-up period [21, 29]. Patients were required to be ≥ 30 years old with established CVD or ≥ 50 years with at least 2 CVD risk factors. Study patients were required to have eGFR > 30 mL/min/1.73 m^2^ (calculated using the MDRD equation); canagliflozin is indicated for T2DM patients with eGFR ≥ 45 mL/min/1.73 m^2^ and contraindicated for patients with eGFR < 30 mL/min/1.73 m^2^ [30].

A total of 6656 (65.6%) CANVAS Program patients had established CVD, most commonly stable coronary artery disease (Fig. 1). The mean eGFR was 76.5 ± 20.5 mL/min/1.73 m^2^, 2039 (20.1%) patients had eGFR < 60, and 8101 (79.9%) had eGFR > 60 mL/min/1.73 m^2^ [21, 31]. The median UACR was 12.3 [6.65, 42.1] mg/g; 7007 (69.8%) patients had UACR < 30 mg/g, 2266 (22.6%) had UACR > 30–300 mg/g, and 760 (7.6%) had UACR > 300 mg/g. Although antihypertensive agent use was not required, patients receiving these drugs were required to have a documented systolic blood pressure higher than 140 mm Hg. Renin-angiotensin-aldosterone system inhibitor (RAASi) use was not required; however, they were used in 8116 (80.0%) patients.

The primary composite CV endpoint (CV death, nonfatal MI, or nonfatal stroke) rate per 1000 patient-years was 26.9 for canagliflozin patients compared to 31.5 for placebo patients (HR = 0.86, 95% CI 0.75–0.97, p = 0.08) (Fig. 2). With regard to secondary outcomes, the HHF rate per 1000 patient-years was 5.5 for canagliflozin patients compared to 8.7 for placebo patients (HR = 0.67, 95% CI 0.52–0.87, p = 0.02). The HHF or CV death rate per 1000 patient-years was 16.3 for canagliflozin patients compared to 20.8 for placebo patients (HR = 0.78, 95% CI 0.67–0.91, p = 0.0015). The composite renal outcome [40% reduction in eGFR sustained for at least two consecutive measures, need for RRT (chronic dialysis, sustained eGFR < 15 mL/min/1.73 m^2^, or kidney transplantation), or renal death] rate per 1000 patient-years was 5.5 in empagliflozin patients compared to 9.0 in placebo patients (HR = 0.6, 95% CI 0.47–0.77, p < 0.001) (Fig. 3) [21].

### The *DECLARE*-*TIMI 58* trial

The third and most recent SGLT2i CVOT, the Dapagliflozin Effect on CardiovascuLAR Events randomized double-blind controlled trial (DECLARE-TIMI 58) assigned 17,160 T2DM patients to 10 mg of dapagliflozin daily or placebo over a median 4.2 [3.9, 4.4] year follow-up period [22]. Males ≥ 55 years or females ≥ 60 years with ≥ 1 CVD risk factor were included in the trial. Study patients were required to have creatinine clearance (CrCl) ≥ 60 mL/min with no specified minimum eGFR [32]. Dapagliflozin is indicated for T2DM patients with eGFR ≥ 45 mL/min/1.73 m^2^ (initially eGFR ≥ 60 but updated to 45 in March 2019) and contraindicated for patients with eGFR < 30 mL/min/1.73 m^2^ [33, 34]. Investigators used the Cockroft-Gault equation to calculate CrCl for the exclusion criteria and the Chronic Kidney Disease Epidemiology Collaboration (CKD-EPI) equation to calculate eGFR when reporting the composite renal outcomes.

In DECLARE-TIMI 58, 6974 (40.6%) patients had established CVD, most commonly stable coronary disease (Fig. 1). The mean eGFR was 85.2 mL/min/1.73 m^2^, 1265 (7.4%) patients had eGFR < 60, and 15,895 (92.6%) had eGFR > 60 mL/min/1.73 m^2^. The median UACR was 13.1 [6.0, 43.6] mg/g; 11,652 (67.9%) patients had UACR < 30 mg/g, 4023 (23.4%) had UACR > 30–300 mg/g, and 1169 (6.8%) had UACR > 300 mg/g. Although ACEi/ARB use was not required, they were used in 13,950 (81.3%) patients.

The primary composite CV endpoint (CV death, nonfatal MI, or nonfatal stroke) occurred in 8.8% of dapagliflozin patients compared to 9.4% of placebo patients (rate per 1000 patient-years = 22.6 vs. 24.2, HR = 0.93, 95% CI 0.84–1.03, p = 0.17) (Fig. 2). With regard to secondary outcomes, HHF occurred in 2.5% of dapagliflozin patients compared to 3.3% of placebo patients (rate per 1000 patient-years = 6.2 vs. 8.5, HR = 0.73, 95% CI 0.61–0.88, p = 0.0008). HHF or CV death occurred in 4.9% of dapagliflozin patients compared to 5.8% of placebo patients (rate per 1000 patient-years = 12.2 vs. 14.7, HR = 0.83, 95% CI 0.73–0.95, p = 0.005). The composite renal outcome [≥ 40% reduction in eGFR to a threshold < 60 mL/min/1.73 m^2^, ESRD (dialysis ≥ 90 days, sustained eGFR < 15 mL/min/1.73 m^2^, or kidney transplantation), or renal/CV death] occurred in 1.5% of dapagliflozin patients compared to 2.8% of placebo patients (rate per 1000 patient-years = 3.7 vs. 7, HR = 0.53, 95% CI 0.43–0.66, p < 0.001) (Fig. 3) [22].

### The *CREDENCE* trial

The Canagliflozin and Renal Events in Diabetes with Established Nephropathy Clinical Evaluation randomized double-blind controlled trial (CREDENCE) assigned 4401 patients with T2DM and CKD to 100 mg of canagliflozin or placebo daily over a 2.62 year median follow-up period [23]. Study patients were required to have eGFR between 30 and 90 mL/min/1.73 m^2^ (calculated using the CKD-EPI equation) and investigators planned to include ~ 60% of patients with eGFR between 30 and 60 mL/min/1.73 m^2^. Additionally, patients were required to have albuminuria, defined as UACR > 300–5000 mg/g. Patients were not required to have prior CVD. Notably, the trial was stopped early as it met the pre-specified efficacy criteria for premature cessation.

In CREDENCE, 2220 (50.4%) patients had established CVD and ~ 16% had a baseline history of HF (Fig. 1). The mean eGFR was 56.2 ± 18.2 mL/min/1.73 m^2^, 2631 (59.8%) patients had eGFR < 60 mL/min/1.73 m^2^, and 1769 (40.2%) had eGFR > 60 mL/min/1.73 m^2^. In striking contrast to the three CVOT trials, the median UACR was 927 [463, 1833] mg/g; 31 (0.7%) patients had UACR < 30 mg/g, 496 (11.3%) had UACR > 30–300 mg/g, 3371 (76.6%) had UACR > 300–3000 mg/g, and 503 (11.4%) had UACR > 3000 mg/g. Stable ACEi/ARB use was required for ≥ 4 weeks prior to randomization and RAASi were used in 4395 (99.9%) patients.

The primary composite renal endpoint [doubling of serum creatinine from baseline (sustained for at least 30 days), ESRD (dialysis, renal transplantation, or sustained eGFR < 15 mL/min/1.73 m^2^), or renal/CV death] occurred in 11.1% of canagliflozin patients compared to 15.4% of placebo patients (rate per 1000 patient-years = 43.2 vs. 61.2, respectively; HR = 0.70, 95% CI 0.59–0.82, p = 0.00001) (Fig. 3). With regard to secondary outcomes, the composite CV outcome (CV death, nonfatal MI, or nonfatal stroke) occurred in 9.9% of canagliflozin patients compared to 12.2% of placebo patients (rate per 1000 patient-years = 38.7 vs. 48.7, HR = 0.80, 95% CI 0.67–0.95, p = 0.01) (Fig. 2). HHF occurred in 4.0% of canagliflozin patients compared to 6.4% of placebo patients (rate per 1000 patient-years = 15.7 vs. 25.3, HR = 0.61, 95% CI 0.47–0.80, p < 0.001). HHF or CV death occurred in 8.1% of canagliflozin patients compared to 11.5% of placebo patients (rate per 1000 patient-years = 31.5 vs. 45.4, HR = 0.69, 95% CI 0.57–0.83, p < 0.001).

## Discussion

### Cardiovascular and renal outcomes

**Fig. 4:**
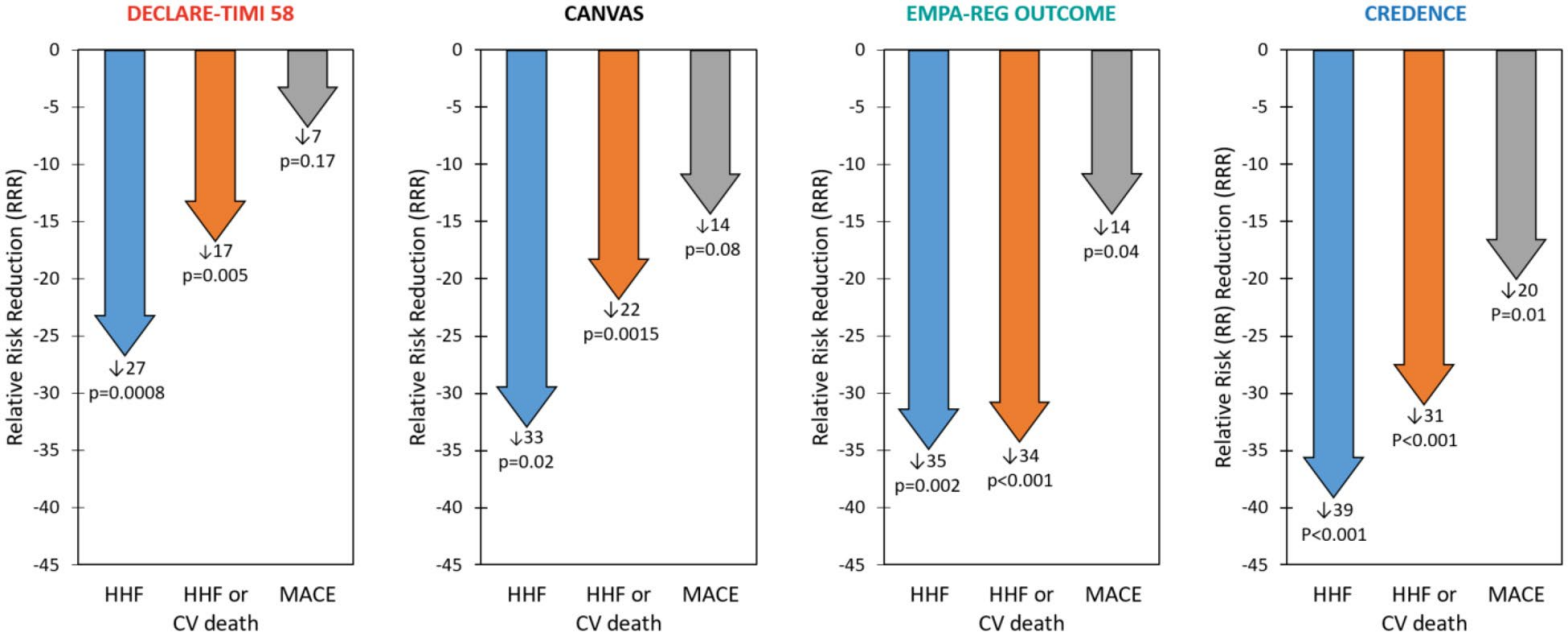
Heart failure hospitalization (HHF), HHF and cardiovascular (CV) death, and major adverse cardiovascular event (MACE) relative risk reductions (RRRs) in the Dapagliflozin Effect on CardiovascuLAR Events (DECLARE-TIMI 58), CANagliflozin CardioVascular Assessment Study (CANVAS) Program, Empagliflozin Cardiovascular Outcome Event Trial in Type 2 Diabetes Mellitus Patients–Removing Excess Glucose (EMPA–REG OUTCOME), and Canagliflozin and Renal Events in Diabetes with Established Nephropathy Clinical Evaluation (CREDENCE) trials. Statistical outcomes displayed as RRR, p-value. RRRs were calculated from hazard ratios

When considering the four SGLT2i trials, we found that overall relative risk reductions for HHF and CV death were externally consistent among the clinical trials (Fig. 4). The relative reductions in HHF were considerably greater than those for ischemic events including nonfatal MI and ischemic stroke. Additionally, the absolute risks of CV events appeared to be more related to baseline renal filtration than the baseline CVD rate (largely comprised of stable coronary artery disease in the patient histories). Finally, when the trial criteria was designed to enroll patients with significant diabetic nephropathy with albuminuria, not only was a compelling reduction in the primary renal composite outcome observed, but the highest rate of CV events was observed as well.

**Fig. 5:**
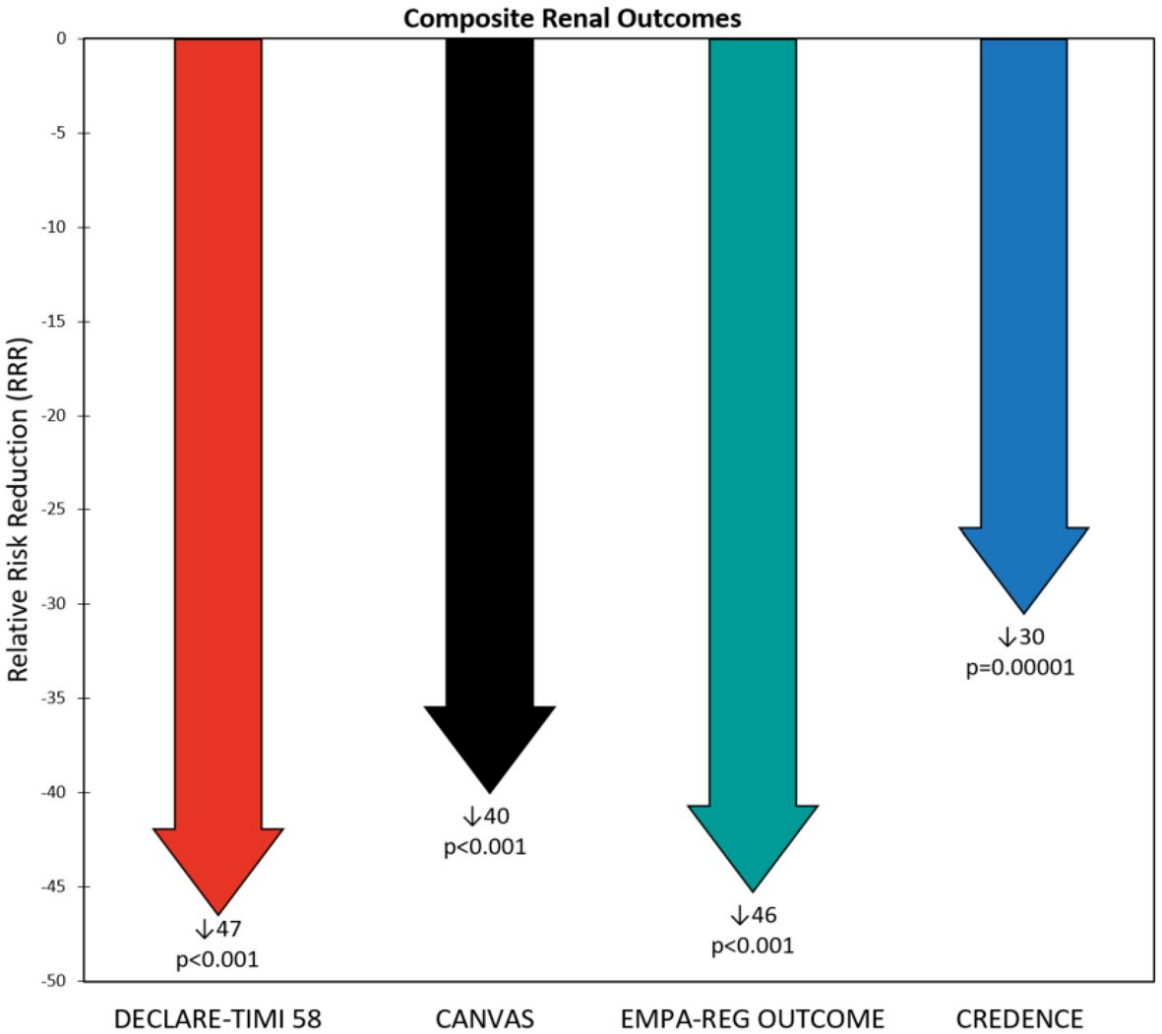
Composite renal outcome relative risk reductions (RRRs) in the Dapagliflozin Effect on CardiovascuLAR Events (DECLARE-TIMI 58), CANagliflozin CardioVascular Assessment Study (CANVAS) Program, Empagliflozin Cardiovascular Outcome Event Trial in Type 2 Diabetes Mellitus Patients–Removing Excess Glucose (EMPA–REG OUTCOME), and Canagliflozin and Renal Events in Diabetes with Established Nephropathy Clinical Evaluation (CREDENCE) trials. Statistical outcomes displayed as RRR, p-value. RRRs were calculated from hazard ratios. Composite renal outcomes defined as follows: DECLARE-TIMI 58: ≥ 40% reduction in estimated glomerular filtration rate (eGFR) to < 60, end-stage renal disease (ESRD) (dialysis ≥ 90 days, transplant or sustained eGFR < 15), or renal/cardiovascular (CV) death; CANVAS: ≥ 40% reduction in eGFR, renal-replacement therapy (RRT) (transplant, chronic dialysis, or sustained eGFR < 15), or renal death; EMPA–REG OUTCOME: doubling of serum creatinine (Cr) with eGFR ≤ 45, RRT, or renal death; CREDENCE: doubling of serum Cr, ESRD (eGFR < 15, dialysis, or renal transplant), renal/CV death

Of the four SGLT2i trials, CREDENCE had the highest CV event rates and DECLARE-TIMI 58 the lowest (Fig. 2). Relative risk reductions (RRRs) varied among the trials, but in general CREDENCE had the largest CV RRRs and DECLARE-TIMI 58 the smallest (Fig. 4). This is consistent with the superior baseline renal filtration function of DECLARE-TIMI 58 patients. CREDENCE had the highest composite renal event rates and DECLARE-TIMI 58 the lowest (Fig. 3). Despite these differences, the relative risk reductions in similar renal composite endpoints were externally consistent among the four trials (Fig. 5).

**Fig. 6:**
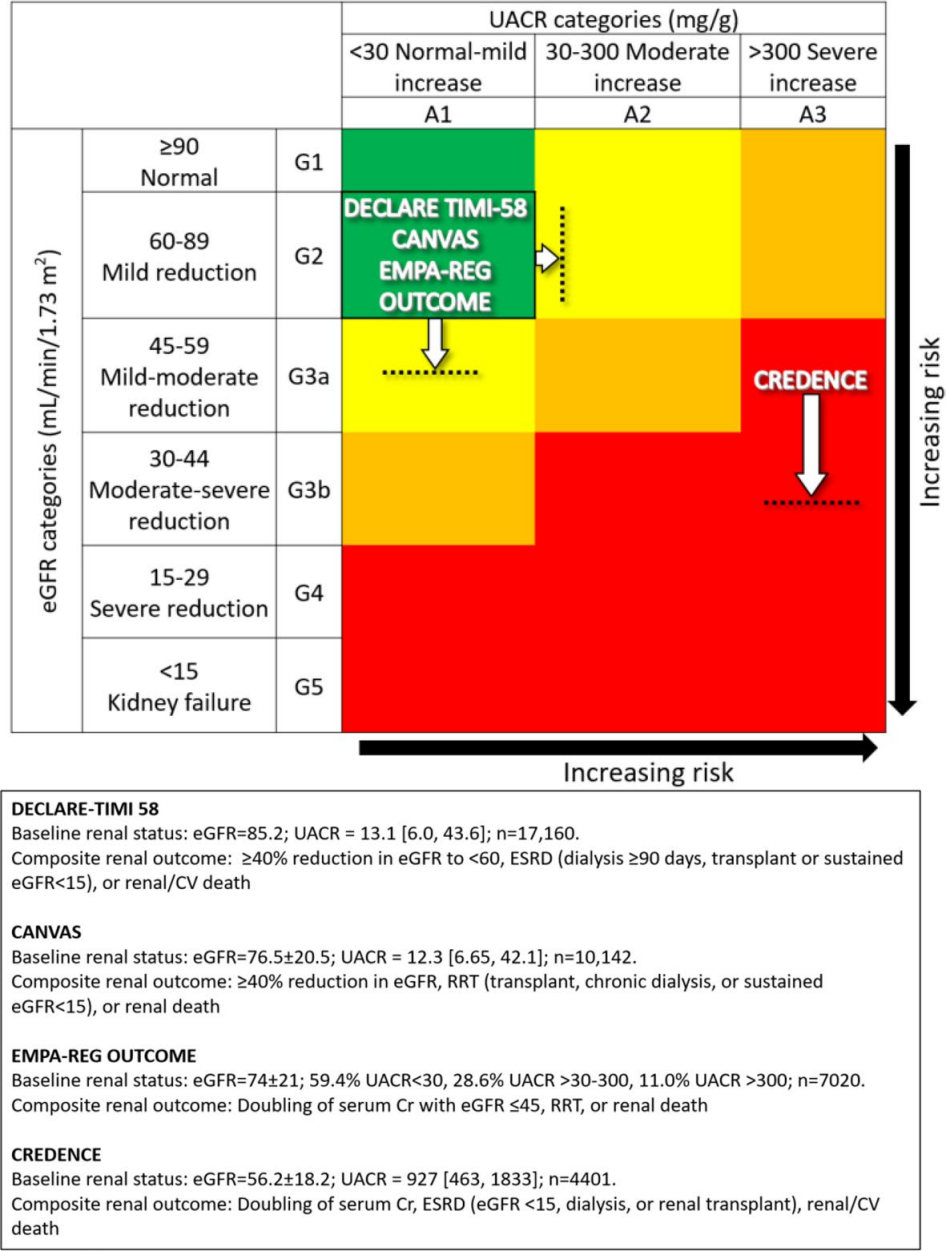
Baseline renal risk and composite renal outcome definitions in the Dapagliflozin Effect on CardiovascuLAR Events (DECLARE-TIMI 58), CANagliflozin CardioVascular Assessment Study (CANVAS) Program, Empagliflozin Cardiovascular Outcome Event Trial in Type 2 Diabetes Mellitus Patients–Removing Excess Glucose (EMPA–REG OUTCOME), and Canagliflozin and Renal Events in Diabetes with Established Nephropathy Clinical Evaluation (CREDENCE) trials. Horizontal dotted lines and white arrows approximate trials averaged mean eGFRs minus 1 pooled standard deviation; vertical dotted lines and white arrows approximate trials’ quartile 3 of UACR. Data displayed as mean eGFR ± standard deviation (where available); median UACR [quartile 1, quartile 3] or percent of study population with UACR < 30, > 30–300, and > 300 (depending on trial). eGFR estimated glomerular filtration rate in mL/min/1.73 m^2^, UACR urinary albumin-creatinine ratio in mg/g (Adapted from Ref. [60])

We described the differences in CV and renal outcomes between the three CVOTs (CANVAS, DECLARE-TIMI 58, EMPA–REG OUTCOME) in a previous review [35]. We argued that the different results of the trials were at least partially attributable to non-standard inclusion criteria, renal filtration function equations, and event definitions rather than inherent differences among the medications. We suspect the same is true when comparing the three CVOTs to CREDENCE—its population had much higher baseline renal risk, and thus experienced more CV and renal outcomes. Specifically, CREDENCE had the lowest mean baseline eGFR (56.2 mL/min/1.73 m^2^) compared to DECLARE-TIMI 58, CANVAS, and EMPA–REG OUTCOME (85.2, 76.5, and 74 mL/min/1.73 m^2^, respectively) (Fig. 1). Most importantly, CREDENCE had the highest degree of albuminuria (median UACR = 927 [463, 1833] mg/g) compared to CANVAS (median UACR = 12.3 [6.65, 42.1] mg/g), DECLARE-TIMI 58 (median UACR = 13.1 [6.0, 43.6] mg/g), and EMPA–REG OUTCOME (median and quartiles 1 and 3 not supplied; 59.4% UACR < 30, 28.6% UACR > 30–300, 11.0% UACR > 300 mg/g). Together, these trials establish the UACR as a risk predictor not only for renal events but also CV outcomes. Figure 6 positions the four trials according to baseline UACR and eGFR; CREDENCE had the highest renal risk and DECLARE-TIMI 58 the lowest. This “heat map” was derived from the Chronic Kidney Disease Prognosis Consortium and the results we have summarized are consistent with the higher absolute renal and CV events observed in the four trials [36].

Note that eGFR is more likely to identify CKD in older patients whereas UACR/albuminuria is more likely to identify it in younger patients [37]. Additionally, albuminuria is an important predictor of CKD progression. We anticipate that some degree of the heterogeneity in cardiorenal outcomes between the trials is accountable to population differences in these two biomarkers.

Baseline renal filtration function appears to play a major role in predicting cardiorenal outcomes, perhaps more so than prior CVD. Even though CREDENCE was not planned as a CVOT and thus only 50.4% of its population had prior CVD (compared to 40.6%, 65.6%, and 99.2% for DECLARE-TIMI 58, CANVAS, and EMPA–REG OUTCOME, respectively), CREDENCE still had, for example, a two-fold increase in MACE compared to DECLARE-TIMI 58. This is supported by findings that SGLT2i decreased CV risk depending on baseline renal filtration function but not prior CVD status—lower function was associated with greater reductions in HHF [38].

### Outcome definitions

We considered variance in trial methodologies as determinants for differences in results. Although the four trials had comparable CV event definitions due to FDA regulatory guidance, their composite renal outcome definitions varied according to sponsor choice (Table 1). For example, DECLARE-TIMI 58 and CREDENCE included CV death while CANVAS and EMPA–REG OUTCOME did not. There were also minor differences in the choice of renal filtration function estimation equation: DECLARE-TIMI 58 and CREDENCE used CKD-EPI to calculate eGFR while the other two trials used the MDRD equation. The CKD-EPI equation is slightly more accurate and precise and is more prognostic for mortality than MDRD [39–42]. We believe these relatively subtle differences in event definitions and estimation of renal filtration function did not play an appreciable role in the trials’ results or interpretation – the most significant factor still appears to be the position of the trials on the renal risk heat map (Fig. 6).

**Table 1:** Renal drug guidelines, entry criteria, mean estimated glomerular filtration rate, and composite outcome definitions in the Dapagliflozin Effect on CardiovascuLAR Events (DECLARE-TIMI 58), CANagliflozin CardioVascular Assessment Study (CANVAS) Program, Empagliflozin Cardiovascular Outcome Event Trial in Type 2 Diabetes Mellitus Patients–Removing Excess Glucose (EMPA–REG OUTCOME), and Canagliflozin and Renal Events in Diabetes with Established Nephropathy Clinical Evaluation (CREDENCE) trials

Trial	FDA indicated guidelines	Study renal entry criteria			Results	
	Minimum recommended eGFR	eGFR minimum	eGFR equation	Additional renal criteria	Mean eGFR	Composite renal outcome
DECLARE-TIMI 58	45	N/A	CKD-EPI	CrCl 60 mL/min (Cockroft-Gault equation)	85.2	≥ 40% reduction in eGFR to < 60, ESRD (dialysis ≥ 90 days, transplant or sustained eGFR < 15), or renal/CV death
CANVAS	45	30	MDRD	N/A	76.5	≥ 40% reduction in eGFR, RRT (transplant, chronic dialysis, or sustained eGFR < 15), or renal death
EMPA–REG OUTCOME	45	30	MDRD	N/A	74	Doubling of serum Cr with eGFR ≤ 45, RRT, or renal death
CREDENCE	45	30	CKD-EPI	UACR 300–5000	56.2	Doubling of serum Cr, ESRD (eGFR < 15, dialysis, or renal transplant), renal/CV death

All eGFRs are in mL/min/1.73 m^2^

*eGFR* estimated glomerular filtration rate, *MDRD* modification of diet in renal disease, *CKD-EPI* chronic kidney disease epidemiology collaboration, *RRT* renal-replacement therapy, *ESRD* end-stage renal disease, *CV* cardiovascular, *CrCl* creatinine clearance, *Cr* creatinine, *UACR* urinary albumin-creatinine ratio in mg/g

### Other notable trial results

Interestingly, the CREDENCE and EMPA–REG OUTCOME placebo groups had similar MACE incidence rates (48.7 and 43.9/1000 patient-years, respectively), despite different baseline UACR and eGFR. We expect this can be attributed to the balance of baseline CVD vs. renal risk: CREDENCE had significantly higher renal risk but only 50.4% prior CVD whereas EMPA–REG had nearly 100% prior CVD.

The composite renal outcome RRR is another intriguing result when comparing the four trials. In a reversal of the trend seen with the other outcomes, CREDENCE had the smallest RRR and DECLARE-TIMI 58 the largest (Fig. 5). We hypothesize that this effect may reflect differences in renal functional reserve (RFR) among the trial participants. RFR is defined as peak eGFR (induced via stress response) minus baseline eGFR and may result from recruiting inactive nephrons or increasing single nephron filtration [43, 44]. RFR has an inverse relationship with CKD stage, decreasing as CKD progresses [45]. Reducing renal hyperfiltration injury in patients with less severe CKD and thus more RFR (i.e., those in the three CVOTs) may yield more robust risk reduction or preventable fraction than in patients with advanced CKD and thus less RFR (i.e., those in CREDENCE). This hypothesis of varying opportunity for prevention of renal filtration function loss needs to be tested formally.

### RAASi use

We considered other sources of confounding for differences among the trials. All four trials had substantial RAASi use: approximately 80% in the three CVOTs and 99.9% in CREDENCE. Thus, we do not believe differential rates of RAASi can explain the contrasts between the trials. Notably, the high rates of RAASi use indicate that the patients were well-treated at baseline. This should ease skepticism about the real-world therapeutic opportunity for SGLT2i, as any benefits due to the SGLT2i can be viewed as being additional to those from RAASi therapy.

### Safety

We balanced our views on efficacy with the safety data. The four trials demonstrated several general safety trends (Tables 2 and 3). SGLT2i were found to be significantly safer than placebo regarding adverse events (AEs) and serious AEs. However, they were generally associated with increased risk of diabetic ketoacidosis and amputation and decreased risk of acute kidney injury. There were no clear trends regarding fractures or urinary tract infections. SGLT2i were significantly associated with increased risk of genital infections; however, this is expected due to the glucosuria promoted by the drugs. Recent research found that SGLT2i are associated with increased risk of Fournier’s gangrene [46, 47]. However, DECLARE-TIMI 58—the only trial of the four prospectively to study this AE—reported that Fournier’s gangrene occurred in 18 (0.2%) of dapagliflozin patients vs. 24 (0.3%) of placebo patients.

**Table 2:** Adverse events in the Dapagliflozin Effect on CardiovascuLAR Events (DECLARE-TIMI 58), CANagliflozin CardioVascular Assessment Study (CANVAS) Program, Empagliflozin Cardiovascular Outcome Event Trial in Type 2 Diabetes Mellitus Patients–Removing Excess Glucose (EMPA–REG OUTCOME), and Canagliflozin and Renal Events in Diabetes with Established Nephropathy Clinical Evaluation (CREDENCE) trials

	DECLARE-TIMI 58					CANVAS				EMPA–REG OUTCOME				CREDENCE					
	DAPA n (%)	Placebo n (%)	Risk^a^	HR (95% CI)	p-value	CANA event rate	Placebo event rate	Risk^a^	p-value	Pooled EMPA n (%)	Placebo n (%)	Risk^a^	p-value	CANA n (%)	Placebo n (%)	CANA event rate	Placebo event rate	Risk^a^	HR (95% CI)
Male genital infection^b^	76 (0.9)	9 (0.1)	+^c^	8.36 (4.19–16.68)^c^	0.001^c^	34.9	10.8	+^c^	< 0.001^c^	166 (5.0)	25 (1.5)	+^c^	< 0.001^c^	28 (0.2)	3 (0.0)	8.4	0.9	+^c^	9.30 (2.83–30.60)^c^
Female genital infection^b^						68.8	17.5	+^c^	< 0.001^c^	135 (10.0)	17 (2.6)	+^c^	< 0.001^c^	22 (0.3)	10 (0.0)	12.6	6.1	+	2.10 (1.00–4.45)
Any AE	N/A	N/A	N/A	N/A	N/A	N/A	N/A	N/A	N/A	4230 (90.2)	2139 (91.7)	−^c^	< 0.001^c^	1784 (8.1)	1860 (0.8)	351.4	379.3	−^c^	0.87 (0.82–0.93)^c^
Serious AE	2925 (34.1)	3100 (36.2)	−^c^	0.91 (0.87–0.96)^c^	< 0.001^c^	104.3	120	−^c^	0.04^c^	1789 (38.2)	988 (42.3)	−^c^	< 0.001^c^	737 (3.3)	806 (0.4)	145.2	164.4	−^c^	0.87 (0.79–0.97)^c^
AE leading to discontinuation	693 (8.1)	592 (6.9)	+^c^	1.15 (1.03–1.28)^c^	0.01^c^	35.5	32.8	+	0.07	813 (17.3)	453 (19.4)	−^c^	< 0.001^c^	N/A	N/A	N/A	N/A	N/A	N/A
Hypoglycemia	58 (0.7)	83 (1.0)	−^c^	0.68 (0.49–0.95)^c^	0.02^c^	50	46.4	+	0.20	1303 (27.8)	650 (27.9)	−	N/A	225 (1.0)	240 (0.1)	44.3	48.9	−	0.92 (0.77–1.11)
UTI	127 (1.5)	133 (1.6)	−	0.93 (0.73–1.18)	0.54	40	37	+	0.38	842 (18.0)	423 (18.1)	−	N/A	245 (1.1)	221 (0.1)	48.3	45.1	+	1.08 (0.90–1.29)
Fracture	457 (5.3)	440 (5.1)	+	1.04 (0.91–1.18)	0.59	15.4	11.9	+^c^	0.02^c^	179 (3.8)	91 (3.9)	−	N/A	67 (0.3)	68 (0.0)	11.8	12.1	−	0.98 (0.70–1.37)
Hyperkalemia	N/A	N/A	N/A	N/A	N/A	6.9	4.4	+	0.10	N/A	N/A	N/A	N/A	151	181	29.7	36.9	−	0.80
Amputation	123 (1.4)	113 (1.3)	+	1.09 (0.84–1.40)	0.53	6.3	3.4	+^c^	< 0.001^c^	N/A	N/A	N/A	N/A	70 (0.3)	63 (0.0)	12.3	11.2	+	1.11 (0.79–1.56)
AKI	125 (1.5)	113 (1.3)	−^c^	0.69 (0.55–0.87)^c^	0.002^c^	3	4.1	−	0.33	45 (1.0)	37 (1.6)	−^c^	< 0.05^c^	86 (0.4)	98 (0.0)	16.9	20	−	0.85 (0.64–1.13)
Breast cancer	36 (0.4)	113 (1.3)	0	1.02 (0.64–1.63)	0.92	3.1	2.6	+	0.65	N/A	N/A	N/A	N/A	8 (0.1)	3 (0.0)	4.1	1.6	+	2.59 (0.69–9.76)
Bladder cancer	26 (0.3)	45 (0.5)	−^c^	0.57 (0.35–0.93)^c^	0.02^c^	1	1.1	−	0.74	N/A	N/A	N/A	N/A	10 (0.0)	9 (0.0)	1.7	1.6	+	1.10 (0.45–2.72)
DKA	27 (0.3)	12 (0.1)	+^c^	2.18 (1.10–4.30)^c^	0.02^c^	0.6	0.3	+	0.14	4 (0.1)	1 (< 0.1)	+	N/A	11 (0.0)	1 (0.0)	2.2	0.2	+^c^	10.80 (1.39–83.65)^c^

*DAPA* dapagliflozin, *CANA* canagliflozin, *EMPA* empagliflozin, *HR* hazard ratio, *CI* confidence interval, *AE* adverse event, *N/A* not available, *UTI* urinary tract infection, *AKI* acute kidney injury, *DKA* diabetic ketoacidosis

^a^Indicates increased (“+”), decreased (“−”), or no difference in (“0”) risk associated with study drug compared to placebo.

^b^DECLARE-TIMI 58 did not differentiate genital infection by sex

^c^Italic indicates statistical significance at the α = 0.05 level

**Table 3:** Risk associated with study drug compared to placebo for adverse events in the Dapagliflozin Effect on CardiovascuLAR Events (DECLARE-TIMI 58), CANagliflozin CardioVascular Assessment Study (CANVAS) Program, Empagliflozin Cardiovascular Outcome Event Trial in Type 2 Diabetes Mellitus Patients–Removing Excess Glucose (EMPA–REG OUTCOME), and Canagliflozin and Renal Events in Diabetes with Established Nephropathy Clinical Evaluation (CREDENCE) trials

	DECLARE-TIMI 58	CANVAS	EMPA–REG OUTCOME	CREDENCE
Male genital infection^a^	+^b^	+^b^	+^b^	+^b^
Female genital infection^a^		+^b^	+^b^	+
Any AE	N/A	N/A	−^b^	−^b^
Serious AE	−^b^	−^b^	−^b^	−^b^
AE causing discontinuation	+^b^	+	−^b^	N/A
Hypoglycemia	−^b^	+	−	−
UTI	−	+	−	+
Fracture	+	+^b^	−	−
Hyperkalemia	N/A	+	N/A	−
Amputation	+	+^b^	N/A	+
AKI	−^b^	−	−^b^	−
Breast cancer	0	+	N/A	+
Bladder cancer	−^b^	−	N/A	+
DKA	+^b^	+	+	+^b^

*AE* adverse event, *N/A* not available, *UTI* urinary tract infection, *AKI* acute kidney injury, *DKA* diabetic ketoacidosis

^a^DECLARE-TIMI 58 did not differentiate genital infection by sex

^b^indicates statistical significance at the α = 0.05 level. “+” = increased risk, “−” = decreased risk, “0” = no difference in risk

### Future potential benefits of SGLT2i

SGLT2i have demonstrated a host of positive effects of interest for future research. In animal models of T2DM female mice, empagliflozin ameliorated kidney injury by promoting glycosuria, and possibly by reducing systemic and renal artery stiffness; canagliflozin attenuated the progression of atherosclerosis, reducing hyperlipidemia, hyperglycemia, and inflammation by lowering the expression of some inflammatory molecules [48, 49]. Of note, the hyperexpressed SGLT1 in cardiomyocytes may represent a potential pharmacological target for cardioprotection [50]. In human studies of T2DM patients, both dapagliflozin and canagliflozin demonstrated beneficial effects on left ventricular diastolic functional parameters [51, 52]. With regard to SGLT2i versus other antihyperglycemic agents, SGLT2i were associated with a reduced risk of HHF compared to dipeptidyl peptidase 4 inhibitors (DPP4i) and canagliflozin was associated with a reduced risk of HHF and a similar risk of MI or stroke compared to DPP4i, glucagon-like peptide-1 agonists, and sulfonylureas [53, 54]. Finally, the EMPA–REG OUTCOME results may be applicable to T2DM patients with a broader CV risk profile, including patients at low risk of CVD [55].

### Class effects

Giugliano et al. [56] studied the three SGLT2i CVOTs and suggested a class effect with regard to HF risk reduction. After reviewing the CVOTs and CREDENCE, we believe this class effect can be expanded to include CV and renal outcomes in general. Note that there is no universal definition of class effect; the closest approximation is the term “class labeling” used by the FDA, which “assumes that all products within a class are closely related in chemical structure, pharmacology, therapeutic activity, and adverse reactions” [57]. With this in mind, we believe there is sufficient evidence of a class effect. Firstly, the SGLT2i have similar molecular structures. Also, though much of their pharmacological methods of action are unknown, we believe one plausible explanation is off-target inhibition of the sodium-proton antiporter/exchanger—a membrane-bound family of channels present in both the heart and kidneys [58, 59]. The four trials are internally consistent, with no particular subgroup benefitting over another and no treatment interactions within any of the trials. The trials are externally consistent with each other, showing reliable cardiorenal benefit (according to baseline risk) and comparable adverse effects. Lastly, the SGLT2i studied have similar known mechanisms of action resulting in losses of glucose and sodium in the urine and reductions in blood pressure and body weight [58]. This proposed pharmacologic class effect would apply more to HHF, CV death, and renal composite events than to the MACE composite outcome, which was not significantly reduced in DECLARE-TIMI 58. Additionally, this class effect is limited to the three SGLT2i we have reviewed in this paper: canagliflozin, dapagliflozin, and empagliflozin. It remains to be seen if it will extend to ertugliflozin, sotagliflozin, and/or other similar agents.

## Conclusions

Dapagliflozin, empagliflozin, and canagliflozin have internally and externally consistent class effects on cardiorenal outcomes and similar safety profiles. Baseline renal filtration function and degree of albuminuria are the most significant indicators of risk for both CV and renal events. Thus, these two factors also anticipate the greatest clinical benefit for SGLT2i.

## Data Availability

Not applicable.

## Abbreviations

ACEi: angiotensin-converting-enzyme inhibitor
AE: adverse event
ARB: angiotensin-receptor blocker
CANVAS: CANagliflozin CardioVascular Assessment Study
CANVAS-R: CANVAS-Renal
CI: confidence interval
CKD: chronic kidney disease
CKD-EPI: chronic kidney disease epidemiology collaboration
CrCl: creatinine clearance
CREDENCE: canagliflozin and renal events in diabetes with established nephropathy clinical evaluation
CV: cardiovascular
CVD: cardiovascular disease
CVOT: cardiovascular outcomes trial
DECLARE-TIMI 58: Dapagliflozin Effect on CardiovascuLAR Events
DPP4i: dipeptidyl peptidase 4 inhibitor(s)
eGFR: estimated glomerular filtration rate
EMPA–REG OUTCOME: Empagliflozin Cardiovascular Outcome Event Trial in Type 2 Diabetes Mellitus Patients–Removing Excess Glucose
ESRD: end-stage renal disease
FDA: United States Food and Drug Administration
HF: heart failure
HHF: heart failure hospitalization
HR: hazard ratio
MDRD: modification of diet in renal disease
MI: myocardial infarction
RAASi: renin–angiotensin–aldosterone system inhibitor
RRR: relative risk reduction
RRT: renal-replacement therapy
SGLT2i: sodium-glucose cotransporter 2 inhibitor(s)
T2DM: type 2 diabetes mellitus
UACR: urinary albumin-creatinine ratio
